# Hooked on the nicotine addiction thesis: a response to DiFranza

**DOI:** 10.1186/1477-7517-10-31

**Published:** 2013-11-18

**Authors:** Reuven Dar, Hanan Frenk

**Affiliations:** 1 Department of Psychology, Tel Aviv University, Ramat Aviv 69978, Israel; 2 The School of Behavioral Sciences, The Academic College of Tel Aviv-Yafo, Tel Aviv, Israel

## Abstract

DiFranza’s rebuttal to our critique of the “Hooked on Nicotine” research program misconstrues our arguments beyond recognition. The grossest misrepresentation of our critique by DiFranza is that we devise (by thwarting science) to rescue “the conventional wisdom” of the “threshold model of nicotine addiction.” In fact, the difference between our positions lies elsewhere: We believe that nicotine is not an addictive drug and that its contribution to the smoking habit is secondary; DiFranza believes that nicotine is so powerfully addictive that novice smokers can lose autonomy over their smoking behavior after one cigarette or even following a single puff. Our review aimed to critically examine the empirical basis of this extreme version of the nicotine “addiction” model. In this brief commentary we illustrate how the commitment to the nicotine “addiction” theory has biased the methodology and the interpretation of the data in “Hooked on Nicotine” research program.

## 

The task of responding to DiFranza’s rebuttal [1] to our critique [2] proved difficult. This is not because we found his arguments compelling, but rather because there is very little relationship between what we actually wrote and how DiFranza cites us. In some cases, he misconstrues our arguments beyond recognition; in others he simply makes up arguments and attributes them to us. In both cases, he proceeds to rebut his fictional version rather than our actual critique.

Here are some examples of arguments misconstrued by DiFranza (see more below): We did not “argue that the data that describe the early onset of nicotine addiction is so different from the conventional wisdom that it is irrelevant” or that “this entire description of the characteristics of tobacco addiction should be ignored by tobacco researchers because it contradicts the DSM.” What we did say was that “findings concerning the speed and ease by which adolescents can become addicted to smoking are invalidated by major conceptual and methodological flaws” that are explicated in our review. We did not claim that the “conclusion that dependence begins quickly is wrong because we [i.e., DiFranza et al] should have marked the onset of ICD dependence at 30 days.” What we did assert is that in the study in which DiFranza et al. *purport to have used ICD criteria*, these criteria were assessed in such a way that “a participant who smoked two cigarette per week (but had planned on smoking only one), spent more time trying to get these two cigarettes than when he used to smoke only one per week and was told by the school nurse that smoking was bad for his health would earn in this study an ICD diagnosis of tobacco dependence. Findings based on such lenient criteria for tobacco dependence are of questionable significance, and again, cannot be compared to findings based on more conservative criteria.”

Here are some examples of arguments we never made: We never said that “prolonged daily use is a prerequisite to addiction.” We do not “argue that the diagnosis of tobacco addiction should be delayed until 3 DSM criteria are present so that a diagnosis will be more meaningful.” We do not “find it impossible to imagine that nicotine might also start to work with the first dose.” We never “argue that only youth who have tried smoking are susceptible [to initiate smoking]” We did not allege that the “hooked on nicotine” research program is “coordinated” in any way. We did not “argue that the data from every other study on early addiction cannot be trusted.” And we certainly did not propose that “data should be declared irrelevant when they contradict popular concepts (!)”.

We shall not endeavor in this response to rectify these misrepresentations, as this would require too much space and would be too redundant with our original review. Instead, we shall focus here on what we perceive as the principal issue that requires clarification and discussion. As for the rest, we invite the interested reader to evaluate our actual critique rather than the made-up version presented in DiFranza’s rebuttal.

The grossest misrepresentation of our critique by DiFranza is that we devise (“by thwarting science”) to rescue “the conventional wisdom” of the “threshold model of nicotine addiction.” Based on this allegation DiFranza proceeds to contest the threshold model that we supposedly endorse and defend in our critique. In reality this debate is not with our actual position but with manufactured straw man hypotheses inexplicably attributed to us. If DiFranza would have as much as skimmed our publications over the past decade, which he eloquently sums up as “a series of papers published by Dar in which he attacks the work of other researchers,” he could not have mistaken our position with “the threshold model”. In fact, our work has consistently called into question the validity of the core “conventional wisdom” in the field of smoking, namely the nicotine “addiction” thesis. For example, we have shown that even deprived smokers do not prefer nicotine to placebo in controlled NRT studies [3] and that the fact that NRT devices are not reinforcing or addictive cannot be accounted for by the nicotine delivery kinetics thesis [4]. We have provided empirical evidence in support of the view that most of the effects of NRT is attributable to placebo [5], that the alleged euphoric effects of nicotine are an experimental artifact [6] and that craving to smoke is largely attributable to psychological factors rather than to nicotine deprivation [7,8]. It is therefore particularly ironic that DiFranza elevates us to the questionable status of defenders of “…cherished theories on nicotine addiction from encroaching reality (p. 4).” The real difference between our positions lies elsewhere: We believe that nicotine is not an addictive drug and that its contribution to the smoking habit is secondary; DiFranza believes that nicotine is so powerfully addictive that novice smokers can lose autonomy over their smoking behavior after one cigarette or even following a single puff. Our review aimed to critically examine the empirical basis of this extreme version of the nicotine “addiction” model. Below, we illustrate how the commitment to the nicotine “addiction” theory has biased the methodology and the interpretation of the data in “hooked on nicotine” research program. We chose to illustrate this bias in relation to two specific methodological questions under contention: (1) Can smokers validly report the causes of their symptoms? And (2) Can nicotine “addiction” be inferred from abstinence-related craving and withdrawal?

## Can smokers validly report the causes of their symptoms?

According to DiFranza, “on general principle Dar and Frenk dismiss outright the idea that smokers can assess their own symptoms.” That is of course not what we wrote. Smokers can report the severity of their sensations and feelings, and that is a valid – in fact the only valid – way to assess those subjective states. What we did assert on principle is that smokers cannot know whether the sensations they experience *are caused by nicotine*. DiFranza goes on to claim that “It wasn’t the taste, or the handling of the cigarette, or the image of smoking they were addicted to, they said it was the nicotine.” However, the participants in these studies *were not asked* what it was they were craving or “addicted to” – so in fact it could have been any behavioral or sensory aspect of smoking. As we review briefly below, there is ample evidence that psychological aspects of smoking are at least as important as nicotine in maintaining the habit. More importantly, even if participants were allowed to choose alternative causes of their “symptoms,” their answers could not have been interpreted as evidence for the real causes of these subjective states. This is not to belittle smokers’ intelligence or insight. As we note in our review, it has been compellingly demonstrated decades ago that people cannot validly report the causes of their own feelings and behaviors [9]. It is common knowledge in psychological research that participants’ reports on the causes of their behaviors or feelings should be interpreted as reasonable inferences rather than at face value. DiFranza disagrees with this assertion. “To argue that smokers cannot attribute their own symptoms to withdrawal is analogous to arguing that women cannot be trusted to determine if their labor contractions are painful (p. 24).” This analogy is obviously false – pain is not a cause of the symptom but the symptom itself. The women respondents in DiFranza’s analogy are not required to make any causal attribution for their pain – they are simply reporting their sensations. DiFranza’s observation that “self-rated addiction shows an excellent correlation with self-rated difficulty quitting (r = .89), and correlates better with all other indicators of dependence than does the DSM” does not indicate that smokers can validly attribute the cause of their sensations to nicotine; it only shows that difficulty quitting is interpreted by smokers as evidence that they are “addicted” (or even synonymous with it).

DiFranza continues to assert that “If alcoholics were asked “what is it about beer that you are addicted to” we would accept an answer of “the alcohol” without requiring that the subject hold a degree in psychopharmacology (p. 24).” DiFranza misses the point that the reason we accept that his “alcoholics” are “addicted” to alcohol rather than to other aspects of beer drinking is *not* because we trust their insight on the matter. It is because we share with those “alcoholics” the theory that alcohol can produce physical dependence and that its psychopharmacologic properties are probably central to their beer drinking habit. Neither “alcoholics” nor smokers have any privileged knowledge of the real factors that cause their wish to continue in the respective destructive behaviors; in both cases they are only making reasonable inferences about these causes. Beer drinker and smokers could not have provided these answers before it was known that cigarettes contain nicotine or that beer contains alcohol. The fact that they provide these answers today is because everyone knows about nicotine and alcohol and their presumed role in smoking and drinking, respectively (even without a degree in psychopharmacology). Whether or not these answers correspond to objective reality has nothing to do with insight – it has to do with the empirical basis of the respective theories; and in this respect alcohol and nicotine are not on equal footing. Alcohol is a substance that may produce physical dependence. It is associated with a drug-specific withdrawal syndrome and with tolerance to its positive effects, which together can account for the tendency of some people to consume progressively more alcoholic drinks. In contrast, as we briefly illustrate below, the case for nicotine as an addictive substance is far from compelling.

## Can nicotine “addiction” be inferred from abstinence-related craving and withdrawal?

The above discussion suggests that the reason it seems obvious to DiFranza that his respondents know that they are “addicted” to nicotine is that he is not considering any alternatives. Throughout his paper, DiFranza uses “nicotine addiction” interchangeably with “tobacco addiction,” suggesting that for him the two are one and the same. For DiFranza, nicotine “addiction” is a disease, diagnosable by “a variety of symptoms that would make quitting more difficult or unpleasant, such as craving, feeling addicted and experiencing withdrawal symptoms (p. 13).” As we document in our review, however, attempts to abstain from many habits, such as the use of pacifier in infants, nail-biting, trichotillomania, gambling and hand-washing, involve craving, feeling addicted and withdrawal symptoms. To determine that such symptoms in smokers reflect the effects of nicotine, it must be shown that they (1) cannot be accounted for by non-drug components of smoking and (2) that they can be produced by the pharmacological properties of nicotine other than in cigarettes. In our opinion, the nicotine “addiction” thesis consistently fails both of these tests. For example, NRTs are only mildly effective in blocking craving and withdrawal symptoms [10]. Denicotinized cigarettes are far more effective and almost indistinguishable from nicotine containing cigarettes [11] in blocking craving and withdrawal symptoms. Moreover, any differences between the two types of cigarettes appear to be due to the contribution of nicotine to the sensory impact of the smoke (via its peripheral receptors) rather than any psychoactive drug effects [12]. Nicotine other than in tobacco does not cause “addiction” [13] and is not self-administered by smokers even following overnight abstinence [14], a finding that cannot be explained by nicotine delivery kinetics [4]. Nicotine antagonists do not precipitate withdrawal even in heavy smokers [15]. Ex-smokers do not become re-“addicted” even following long term exposure to nicotine in the absence of tobacco [13]. We believe that because the nicotine “addiction” theory fails these essential tests, it cannot account for the prevalence of the smoking habit or for the difficulty many smokers experience when they attempt to reduce or quit smoking. Notably, the force of this commulative evidence has recently led Karl Fagerstrom [16] to suggested to rename the “Fagerstrom Test for Nicotine Dependence” the Fagerstrom to Cigarette Dependence.”^a^

In our opinion, this situation calls for an alternative theory that places sensory and behavioral aspects of smoking on central stage. Such a theory would account for craving and withdrawal from smoking in behavioral and psychological terms rather than as symptoms of drug “addiction”. This view is clearly very different from the one expressed by DiFranza and the “hooked on nicotine” research program. We believe that is also reflects more faithfully the current state of our knowledge.

## Endnote

^a^It would be fitting to summarize the evidence for the “addictive” properties of e-cigarettes. However, when this paper was originally submitted (about 3 years ago) there was very little available research on the topic. Even today, evidence relevant to this important question is still scarce.

## Competing interests

RD and HF have received fees for consulting to Imperial Tobacco Group PLC. However, all their research, including this review, is supported exclusively by academic funds.

## Authors’ contributions

RD and HF wrote the manuscript together. All authors read and approved the final manuscript. Both authors read and approved the final manuscript.
